# Automated postoperative SLO quantification across clinically recorded cataract subtype groups: an exploratory cross-sectional study

**DOI:** 10.3389/fmed.2026.1896907

**Published:** 2026-09-10

**Authors:** Yixue Yin, Xinran Guo, Shuo Xu, Xiuyan Zhang, Hongsheng Bi, Jike Song

**Affiliations:** 1 Shandong University of Traditional Chinese Medicine, Jinan, China; 2 Affiliated Eye Hospital of Shandong University of Traditional Chinese Medicine, Jinan, China; 3 Shandong Provincial Key Laboratory of Integrated Traditional Chinese and Western Medicine for Prevention and Therapy of Ocular Diseases, Jinan, China; 4 Medical College of Optometry and Ophthalmology, Shandong University of Traditional Chinese Medicine, Jinan, China

**Keywords:** age-related cataract, artificial intelligence, cataract subtype, posterior segment imaging, scanning laser ophthalmoscopy

## Abstract

**Objective:**

To explore the application of automated quantitative scanning laser ophthalmoscopy analysis to early postoperative images and to characterize group-level variation in the resulting measurements across patients grouped according to clinically recorded age-related cataract subtype.

**Methods:**

This retrospective observational imaging study included 716 eyes from 571 patients with age-related cataract who underwent phacoemulsification combined with intraocular lens implantation between August 2023 and August 2025. Early postoperative scanning laser ophthalmoscopy (SLO) images were analyzed using EVisionAI version 4.0 as a standardized automated quantitative image-analysis tool. Eyes were grouped as nuclear cataract (394 eyes), cortical cataract (217 eyes), or posterior subcapsular cataract (PSC; 105 eyes) according to routine preoperative slit-lamp records. Standardized LOCS III grading and cohort-specific validation of EVisionAI against manual annotations were not available in this retrospective dataset. Automated image-derived parameters included tessellated fundus-related features, optic disc morphology, peripapillary atrophy (PPA)-related parameters, and retinal vascular parameters. Patient-level baseline characteristics were compared using conventional statistical tests, as appropriate. Eye-level comparisons of automated image-derived SLO parameters were performed using generalized estimating equations to account for inter-eye correlation, with Wald χ^2^ statistics reported. Multiple comparisons were adjusted using the Benjamini–Hochberg false discovery rate procedure. Multivariable GEE models adjusted for age, sex, axial length, and cataract subtype were used to evaluate associations with selected imaging parameters. Standardized effect-size, age-overlap, and single-eye sensitivity analyses were also performed.

**Results:**

After false discovery rate correction, multiple automated image-derived fundus parameters differed significantly among the retrospectively defined cataract subtype groups, including cup-to-disc ratio parameters, optic disc morphology, PPA-related parameters, and retinal vascular parameters. Compared with PSC eyes, nuclear and cortical cataract eyes showed relatively higher cup-to-disc ratio-related parameters and larger PPA-related parameters, whereas PSC eyes showed relatively greater retinal vessel length. Across age groups, tessellated fundus density, macular tessellated fundus density, optic disc-related tessellated fundus parameters, and PPA-related parameters increased significantly with age. Among the 14 contrasts involving the seven principal outcomes, standardized effect estimates were small to moderate (absolute standardized β, 0.258–0.619), and none met the descriptive threshold for a large effect. In multivariable GEE models, age remained associated with multiple tessellated fundus- and PPA-related parameters after adjustment for sex, axial length, and cataract subtype. Axial length also remained associated with optic disc tilt angle, PPA-related parameters, and tessellated fundus parameters. The age-overlap and single-eye sensitivity analyses showed generally consistent directions for the principal associations, although one optic disc tilt-angle contrast was attenuated after age restriction.

**Conclusion:**

Automated quantitative SLO analysis was completed in 716 of 821 initially available early postoperative images. Group-level differences were observed across clinically recorded cataract subtype groups, but these findings were exploratory and may reflect non-standardized subtype assignment, unmeasured spherical-equivalent refractive error, age, axial length, image characteristics, and other confounding. They should not be interpreted as subtype-specific structural phenotypes or as evidence of diagnostic, prognostic, or clinical decision utility. Prospective studies with standardized cataract grading and cohort-specific algorithm validation are required.

## Introduction

Age-related cataract remains a major cause of visual impairment worldwide, and phacoemulsification combined with intraocular lens implantation is the standard surgical treatment (1, 2). With the transition of cataract surgery from a sight-restoring procedure to a refractive and quality-of-vision procedure, accurate perioperative assessment has become increasingly important (3). Although removal of lens opacity is the primary determinant of visual improvement, postoperative visual recovery may also be influenced by pre-existing retinal, choroidal, optic disc, or vascular abnormalities that are not fully appreciated before surgery (4).

In clinical practice, dense lens opacity may limit fundus visualization and reduce the sensitivity of routine preoperative assessment (5). Optical coherence tomography and ocular ultrasonography can provide useful structural information, but they do not always provide wide-field, multidimensional, and quantitatively standardized characterization of the posterior pole and peripapillary region. Scanning laser ophthalmoscopy (SLO), particularly when combined with automated image analysis, offers an opportunity to quantify fundus features over a broader field of view and to reduce subjective variation in image interpretation (6).

Different clinically recorded cataract subtype groups may differ in age distribution, ocular biometry, refractive status, media opacity characteristics, image-acquisition conditions, and other measured or unmeasured characteristics (7). In the present study, cataract subtype was therefore treated as a retrospective clinical grouping variable rather than as a presumed biological determinant of posterior segment structure. Any observed between-group differences could reflect age, axial length, unmeasured spherical-equivalent refractive error, ocular anatomy, image-acquisition factors, or other confounding and should not be interpreted as subtype-specific structural phenotypes, postoperative prognosis, clinical utility, or causal relationships.

The present study was designed as a retrospective observational imaging analysis. We compared automated quantitative SLO parameters among patients grouped according to clinically recorded nuclear, cortical, or posterior subcapsular cataract subtype and further examined their associations with age and axial length. Cataract subtype was treated as an observational grouping variable rather than as a presumed cause of posterior segment remodeling. The analyses were intended to identify between-group structural variation and to generate hypotheses for future prospective studies incorporating standardized lens grading and comprehensive refractive assessment.

## Materials and methods

### Study design and participants

This retrospective observational imaging study included 716 eyes of patients with age-related cataract who underwent phacoemulsification combined with intraocular lens implantation at the Affiliated Eye Hospital of Shandong University of Traditional Chinese Medicine between August 2023 and August 2025. The study adhered to the Declaration of Helsinki and was approved by the institutional ethics committee (Ethics approval No. HEC-KS-2024035KY). The requirement for written informed consent was waived by the ethics committee because this retrospective study used anonymized clinical and imaging data collected during routine care.

The inclusion criteria were: (1) diagnosis of age-related cataract; (2) age ≥50 years; (3) no family history of hereditary ocular disease; (4) availability of routine ophthalmic examination data; and (5) availability of early postoperative SLO imaging for image-quality assessment. The exclusion criteria were: (1) cataract secondary to diabetes, congenital disease, intoxication, trauma, or other non-age-related causes; (2) ocular hypertension requiring medication or a history of elevated intraocular pressure, including glaucoma or steroid-related ocular hypertension; (3) previous pars plana vitrectomy or retinal reattachment surgery; (4) retinal vascular occlusion, diabetic retinopathy, age-related macular degeneration, pathologic myopia; (5) psychiatric or systemic conditions preventing cooperation with ophthalmic examinations; and (6) incomplete clinical or imaging records or poor-quality fundus images.

Because this was a retrospective study, formal Lens Opacities Classification System III (LOCS III) grading was not consistently available. Cataract subtype was determined according to the predominant opacity pattern documented in the preoperative slit-lamp examination records. Eyes were categorized into nuclear cataract (*n* = 394), cortical cataract (*n* = 217), and posterior subcapsular cataract (PSC; *n* = 105). In eyes with mixed cataract features, classification was based on the predominant opacity type recorded by the attending cataract specialist. Eyes with ambiguous or insufficient subtype documentation were excluded.

The number and proportion of eyes with mixed morphology could not be reliably reconstructed because the available analytic records generally retained the final predominant-subtype label rather than standardized scores for each opacity component. The exact number of clinicians contributing to the original subtype documentation during the study period was also not recoverable from the analytic dataset. No centralized rereview, duplicate grading, or inter-rater reliability assessment was performed.

### Ophthalmic examinations and surgery

All eyes underwent routine perioperative ophthalmic examinations, including uncorrected visual acuity, best corrected visual acuity (BCVA), intraocular pressure, slit-lamp biomicroscopy, dilated fundus examination when possible, IOL Master biometry, ophthalmic B-scan ultrasonography, corneal endothelial microscopy, and macular optical coherence tomography (OCT). BCVA was recorded as decimal visual acuity. Routine OCT examinations were available for clinical screening, but standardized quantitative OCT variables were not consistently retrievable for all included eyes and were not included in the planned analytic dataset. All surgeries were performed by the same experienced surgeon using standard phacoemulsification combined with intraocular lens implantation. Information on IOL model and power, surgical duration, cumulative dissipated energy, intraoperative difficulty, posterior capsule or iris manipulation, and standardized postoperative inflammation grading was not uniformly available. Consequently, these surgical and postoperative variables could not be incorporated into the multivariable models.

Postoperative images were acquired using a California ultra-widefield scanning laser ophthalmoscope (SLO, Optos plc, Dunfermline, United Kingdom) in pseudocolor mode, with a nominal field of view of up to 200°. Postoperative imaging was performed at approximately 7 days because this corresponded to the routine early postoperative visit at our institution and permitted fundus imaging after removal of lens opacity. Imaging was undertaken only after clinical confirmation of a clear cornea, adequate pupil dilation, and a clinically stable anterior segment. Image acquisition was supervised by trained ophthalmic technicians. Patients were instructed to maintain fixation on the device target, and image acquisition was repeated when substantial motion blur or decentration was observed. Images with persistent fixation-related blur, incomplete optic disc or macular visualization, or insufficient image quality were excluded. Representative accepted automated outputs and examples of image-quality or segmentation failure are shown in Supplementary Figure 2. Quantitative fixation stability was not recorded. The present analysis focused on automated image-derived quantitative fundus imaging parameters rather than surgical outcome comparison.

### Automated fundus image analysis

Quantitative analysis of scanning laser ophthalmoscopy images was performed using EVisionAI version 4.0 (registered product model: EVISION-EG; Jiangxi Yiwei Artificial Intelligence Technology Co., Ltd., Ganzhou, Jiangxi, China). EVISION-EG is registered in China as a Class II medical device under Registration No. 20202210553. According to the registration certificate, the registered product is intended for the management, viewing, transmission, and storage of fundus examination images in JPG format and associated data and does not include automated diagnosis.

In the present study, EVisionAI version 4.0 was used in its commercially available configuration for automated optic disc and macular localization, retinal region extraction, segmentation of predefined fundus structures, and quantitative feature extraction. No model development, retraining, fine-tuning, or modification was performed using the present dataset. The automated quantitative outputs evaluated in this study were used for research analysis and were not interpreted as regulatory-approved diagnostic or prognostic outputs. All AI-generated outputs were reviewed by a trained ophthalmic grader for gross segmentation failure before statistical analysis. Images with poor quality, incomplete optic disc or macular visualization, or gross segmentation failure were excluded. Manual correction of segmentation outputs was not performed. Of 821 initially available postoperative SLO images, 105 were excluded before statistical analysis because of poor image quality, incomplete optic disc or macular visualization, or gross segmentation failure, leaving 716 eyes for final analysis. The study flow is shown in Supplementary Figure 1, and exclusion rates across cataract subtypes are provided in Supplementary Table 2. The excluded images were not included in any statistical analysis, and exclusion was performed before group comparisons. EVisionAI uses a deep learning-based object detection network (ResNet-50) for automatic localization of the optic disc and macula, with the center of the macular detection box defined as the macular center (8, 9). Subsequently, deep learning-based segmentation algorithms were used for automatic identification and segmentation of tessellated fundus regions, the optic cup, optic disc, peripapillary atrophy (PPA), and retinal vessels, followed by quantitative parameter extraction.

In the present study, a circular region centered on the macula with a radius of 5.5 mm was defined as the primary analysis region. EVisionAI automatically measured and calculated tessellated fundus-related parameters, including tessellated fundus density, macular tessellated fundus density, optic disc and macular tessellated fundus density, and peripapillary tessellated fundus density. Optic nerve head-related parameters included vertical cup-to-disc ratio, area cup-to-disc ratio, cup diameter, cup area, disc diameter, disc area, optic disc tilt angle, disc circularity, and other optic disc morphological parameters. Peripapillary atrophy-related parameters included PPA height, width, area, and their corresponding ratios relative to optic disc structures. Vascular parameters included retinal vascular fractal dimension and retinal vessel length. Patient-specific ocular magnification correction based on axial length, keratometry, or refractive error was not applied before quantitative parameter extraction. The physical units reported by the software were used as provided, without individualized biometric recalibration. Accordingly, angular parameters, such as cup-to-disc ratios, structural ratios, circularity measures, and disc tilt angle, were distinguished from absolute linear and area measurements reported in μm, mm, or μm^2^.

The reproducibility and reliability of EVisionAI in fundus structure segmentation, parameter extraction, and quantitative analysis have been validated in previous studies, and the platform has been applied in structural analysis of multiple fundus diseases, including high myopia, diabetic retinopathy, and retinal vascular disorders (10–13). However, these previous applications do not constitute validation in the present postoperative cataract cohort (14). No manual annotation-based validation, test–retest assessment, or inter-grader reliability analysis was performed in the current study. Therefore, the present study used EVisionAI as a fixed quantitative image-analysis tool rather than as a diagnostic or prognostic AI model.

### Statistical analysis

Statistical analyses were performed using SPSS version 29.0 (IBM Corp., Armonk, NY, USA) and R version 4.6.0 (R Foundation for Statistical Computing, Vienna, Austria). The primary descriptive analyses, baseline comparisons, omnibus eye-level generalized estimating equation analyses, and single-eye sensitivity analyses were conducted using SPSS. The adjusted multivariable GEE models reported in Table 4 were generated in R using the geepack package version 1.3.13 and the dplyr package version 1.2.1. For the seven principal outcomes, the full-cohort estimates in Supplementary Table 4 and the original-unit estimates in Supplementary Table 5 were extracted from the same fitted model objects used for Table 4. The standardized-outcome models for Supplementary Table 5 and the *post hoc* age-overlap sensitivity models were also fitted in R using the same covariate structure.

Continuous variables were summarized as mean ± standard deviation or median with interquartile range, depending on their distributions. Categorical variables were summarized as frequencies and percentages. All statistical tests were two-sided. A *P*-value < 0.05 was considered statistically significant unless otherwise specified.

Baseline demographic and clinical characteristics were analyzed at the patient level. Comparisons among cataract subtype groups were performed using the chi-square test or Fisher’s exact test for categorical variables and one-way analysis of variance or the Kruskal–Wallis H test for continuous variables, as appropriate. When an overall baseline comparison was statistically significant, *post hoc* pairwise comparisons were performed using Dunn’s test with Bonferroni adjustment for non-normally distributed continuous variables.

Because some patients contributed both eyes, the primary eye-level analyses of automated image-derived SLO parameters were performed using generalized estimating equations to account for inter-eye correlation. Patient identification was specified as the clustering variable, and an exchangeable working correlation structure was used. For continuous imaging outcomes, generalized estimating equation models with a Gaussian distribution, identity link function, and robust sandwich standard errors were fitted. Overall effects of cataract subtype and age category were assessed using Wald χ^2^ statistics.

For age-stratified descriptive analyses, age categories of ≤69, 70–79, and ≥80 years were used to present decade-related patterns and were not intended to represent biological thresholds. Age was modeled as a continuous variable in the primary multivariable analyses. Automated image-derived imaging parameters were descriptively reported as median with interquartile range, whereas inferential comparisons were based on generalized estimating equation models.

The Benjamini–Hochberg false discovery rate procedure was applied separately across the 18 outcome-level omnibus tests for cataract subtype and the 11 outcome-level omnibus tests for age group. When an overall subtype effect was statistically significant, pairwise comparisons for selected representative outcomes were performed using estimated marginal means from the generalized estimating equation models, with adjustment for multiple comparisons as reported in Supplementary Table 1.

To evaluate adjusted associations with selected automated image-derived SLO parameters, multivariable generalized estimating equation models were constructed with continuous age, sex, axial length, and cataract subtype as covariates. Covariates were selected *a priori* on the basis of biological relevance and consistent availability in the retrospective dataset. Spherical-equivalent refractive error was not uniformly available in a methodologically comparable form and was therefore not included in the multivariable models. It was considered an unmeasured potential confounder, particularly when interpreting PPA-, tessellated fundus-, and retinal vascular-related measurements. Posterior subcapsular cataract was used as the reference category for cataract subtype, and male sex was used as the reference category for sex. Regression coefficients, 95% confidence intervals, Wald χ^2^ statistics, and nominal *P*-values were reported. *P*-values from these primary multivariable models were not adjusted for multiple testing. The models were interpreted as association models rather than predictive or causal models.

To facilitate comparison of effect magnitude across imaging outcomes expressed in different units, an additional standardized effect-size analysis was performed in R. Each of 18 selected subtype-associated imaging outcomes was standardized to a z score using the mean and standard deviation of the full analytic cohort. For each outcome, two generalized estimating equation models were fitted using the same covariate structure: one using the original-unit outcome and one using the standardized outcome. Both models included continuous age, sex, axial length, and cataract subtype, with patient identification specified as the clustering variable, an exchangeable working correlation structure, and robust sandwich standard errors. The standardized subtype coefficients represent adjusted between-group differences expressed in standard-deviation units. Original-unit and standardized coefficients with 95% confidence intervals are reported together in Supplementary Table 5. Benjamini–Hochberg false discovery rate correction was applied across the 36 evaluated subtype contrasts. Effect-size categories were used only as descriptive statistical guides and were not interpreted as validated thresholds of clinical importance.

To assess the potential influence of baseline age imbalance, a *post hoc* age-overlap sensitivity analysis was performed in R. The analysis was restricted to patients aged 61–78 years, corresponding to the intersection of the subtype-specific 5th–95th percentile age ranges. The principal subtype association models were refitted in the restricted cohort using generalized estimating equations with adjustment for continuous age, sex, and axial length. Patient identification was specified as the clustering variable, and an exchangeable working correlation structure with robust sandwich standard errors was used. Posterior subcapsular cataract and male sex were used as the reference categories. Benjamini–Hochberg false discovery rate correction was applied across the 14 evaluated subtype contrasts involving seven principal imaging outcomes: vertical cup-to-disc ratio, vertical cup diameter, optic disc tilt angle, PPA arc height, PPA arc width, PPA area, and retinal vessel length. This analysis was intended to reduce extrapolation beyond the overlapping age distributions and was not interpreted as establishing independent or causal effects of cataract subtype.

A single-eye sensitivity analysis was performed to assess whether the primary findings were materially influenced by inter-eye correlation. One eye per patient was selected according to a predefined rule. The first operated eye was selected; when both eyes were operated on during the same period, one eye was selected at random. Multivariable linear regression models including age, sex, axial length, and cataract subtype were then fitted in the single-eye dataset. Regression coefficients, 95% confidence intervals, adjusted R^2^ values, and nominal *P*-values were reported. *P*-values from the single-eye sensitivity models were not adjusted for multiple testing.

Missing data were handled using complete-case analysis for each outcome-specific model.

## Results

### Baseline characteristics

**FIGURE 1:**
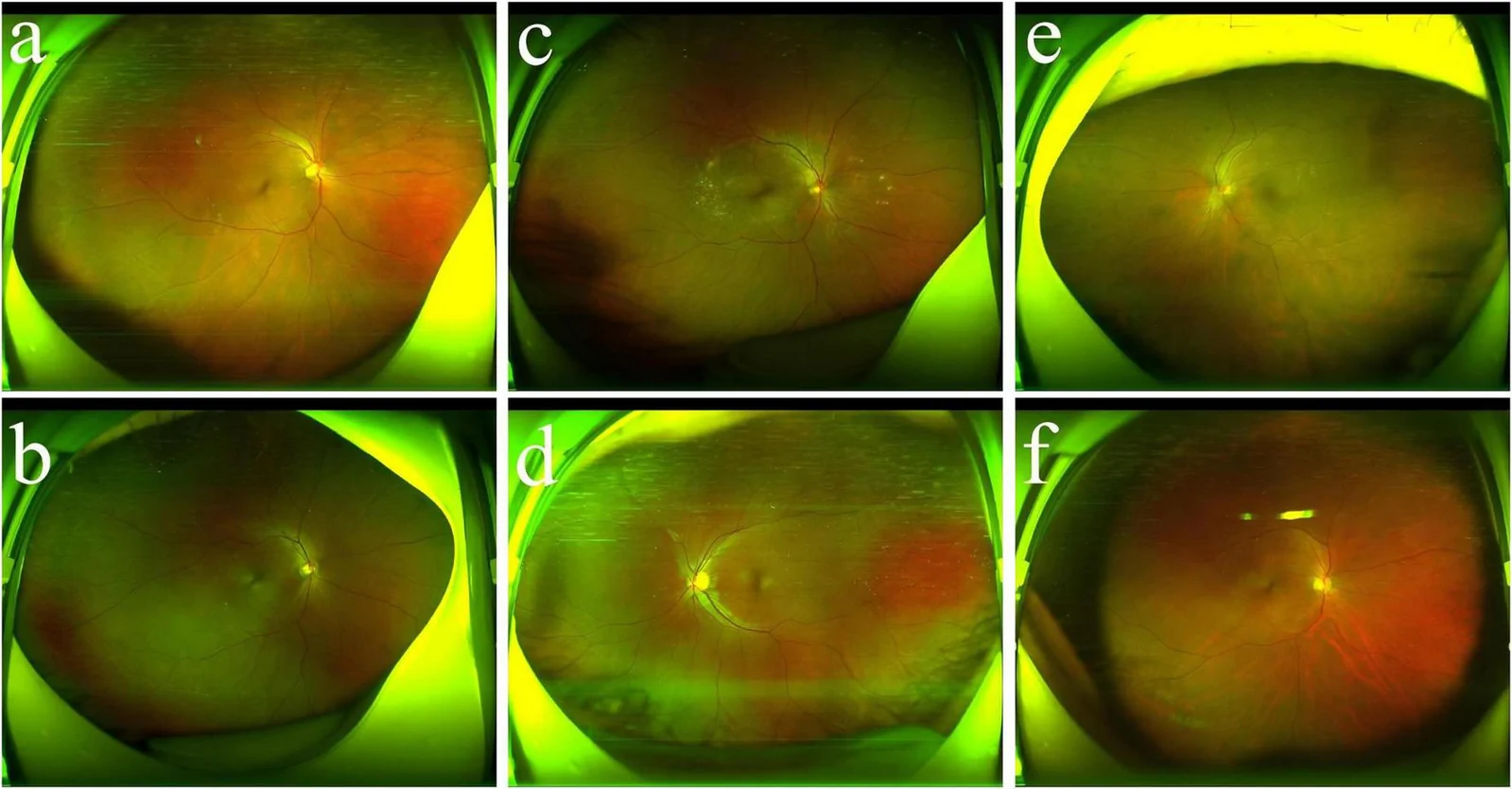
Representative early postoperative SLO images from eyes with nuclear cataract **(a,b)**, cortical cataract **(c,d)**, and posterior subcapsular cataract **(e,f)**. These examples illustrate the range of postoperative SLO image appearances included in the quantitative analysis and are not intended to imply diagnostic differences at the individual-image level.

A total of 571 patients were included in the analysis: 321 patients in the nuclear cataract group, 185 patients in the cortical cataract group, and 65 patients in the posterior subcapsular cataract group (Figure 1). The sex distribution did not differ significantly among the three groups (χ^2^ = 1.092, *P* = 0.579). Median age differed significantly among groups (*H* = 8.776, *P* = 0.012). Patients in the posterior subcapsular cataract (PSC) group tended to be younger than those in the nuclear cataract group, whereas the remaining pairwise comparisons were not statistically significant. Median axial length did not differ significantly among groups (*H* = 5.785, *P* = 0.055). Median BCVA differed significantly among groups (*H* = 26.955, *P* < 0.001), and pairwise comparisons demonstrated significant differences among all three groups, with the PSC group showing the lowest decimal BCVA, indicating poorer preoperative visual acuity. Baseline characteristics are summarized in Table 1.

**TABLE 1:** Baseline characteristics of patients with different cataract subtypes.

Variable	Nuclear group (n = 321)	Cortical group (*n* = 185)	PSC group (*n* = 65)	Statistic	*P*-value
Sex, *n* (%)				χ^2^ = 1.092	0.579
Male	172 (53.6)	108 (58.4)	36 (55.4)		
Female	149 (46.4)	77 (41.6)	29 (44.6)		
Age, years	72.0 (69.0, 76.5)	72.0 (67.0, 76.0)	70.0 (66.5, 74.5)	*H* = 8.776	0.012
Axial length, mm	23.21 (22.58, 23.66)	23.15 (22.56, 23.58)	23.46 (22.83, 23.95)	*H* = 5.785	0.055
BCVA	0.40 (0.25, 0.60)	0.50 (0.40, 0.80)	0.30 (0.15, 0.50)	*H* = 26.955	<0.001

### Automated image-derived fundus parameters across cataract subtypes

In eye-level GEE analyses accounting for inter-eye correlation, multiple automated image-derived fundus parameters differed significantly among cataract subtypes, including cup-to-disc ratio parameters, optic disc shape and tilt-related parameters, peripapillary atrophy (PPA)-related parameters, and vascular parameters. Although these differences reached statistical significance, the absolute between-group differences were generally modest. Overall, the nuclear cataract group tended to show relatively higher cup-to-disc ratio-related parameters, whereas the posterior subcapsular cataract (PSC) group showed relatively lower values in selected optic disc structural parameters and relatively greater vessel length. These findings indicate group-level variation in automated image-derived measurements across the clinically recorded subtype groups, but they do not establish subtype-specific structural phenotypes or causal effects. Overall group comparisons are shown in Table 2. Pairwise comparisons for selected representative parameters are provided in Supplementary Table 1. Because patient-specific ocular magnification correction was unavailable, between-group differences in absolute linear and area measurements should be interpreted with greater caution than differences in dimensionless or angular parameters.

**TABLE 2:** Representative automated image-derived SLO parameters showing structural differences across cataract subtypes.

Variable	Nuclear (*n* = 394)	Cortical (*n* = 217)	PSC (*n* = 105)	Wald χ^2^ (df = 2)	*P*-value	FDR-adjusted *P*-value
Vertical cup-to-disc ratio	0.55 (0.48, 0.61)	0.54 (0.48, 0.60)	0.54 (0.46, 0.60)	10.414	0.005	0.011
Area cup-to-disc ratio	0.29 (0.23, 0.37)	0.29 (0.23, 0.37)	0.29 (0.21, 0.36)	10.023	0.007	0.011
Vertical cup diameter (μm)	896.00 (700.00, 1106.00)	854.00 (686.00, 1127.00)	770.00 (539.00, 1008.00)	9.934	0.007	0.011
Cup area (μm^2^)	649152.00 (399007.00, 946582.00)	630336.00 (372596.00, 1032630.00)	465010.00 (234250.00, 780250.00)	6.905	0.032	0.034
Vertical-to-horizontal disc ratio	0.97 (0.93, 1.02)	0.98 (0.93, 1.04)	0.94 (0.88, 1.02)	22.630	<0.001	0.003
Disc tilt angle (°)	30.86 (13.14, 47.91)	33.72 (14.86, 50.42)	18.10 (2.25, 41.89)	7.030	0.030	0.034
PPA arc height (μm)	1638.00 (1291.50, 2114.00)	1596.00 (1288.00, 2044.00)	1526.00 (969.50, 2247.00)	19.416	<0.001	0.003
Ratio of PPA arc height to vertical disc diameter	1.04 (0.88, 1.18)	1.03 (0.84, 1.15)	0.99 (0.67, 1.21)	25.601	<0.001	0.003
PPA area (μm^2^)	555464.00 (318843.00, 1085542.00)	615636.00 (307426.00, 992934.00)	245000.00 (153136.00, 460894.00)	23.014	<0.001	0.003
Vessel length (mm)	1460.41 (1257.44, 1837.72)	1518.85 (1238.08, 1868.14)	1676.58 (1299.22, 2035.37)	10.845	0.004	0.008

Values are presented as median (interquartile range). Eye-level comparisons were performed using generalized estimating equations to account for inter-eye correlation. Multiple comparisons were adjusted using the Benjamini–Hochberg false discovery rate procedure. The complete set of automated image-derived parameters is provided in Supplementary Table 3. PSC, posterior subcapsular cataract; PPA, peripapillary atrophy; SLO, scanning laser ophthalmoscopy; FDR, false discovery rate. Patient-specific ocular magnification correction was not applied. Absolute measurements expressed in μm, mm, or μm^2^ should therefore be interpreted with caution.

### Automated image-derived fundus parameters across age groups

In age-stratified eye-level GEE analyses, multiple automated image-derived fundus parameters differed significantly, including tessellated fundus-related parameters, peripapillary atrophy (PPA)-related parameters, and selected vascular parameters. Although these differences reached statistical significance, the absolute between-group differences were generally modest. Overall, tessellated fundus density, macular tessellated fundus density, optic disc-related tessellated fundus density, and PPA-related parameters tended to increase with age. Results are summarized in Table 3.

**TABLE 3:** Automated image-derived SLO parameters across age groups.

Variable	≤69 years (*n* = 260)	70–79 years (*n* = 373)	≥80 years (*n* = 83)	Wald χ^2^ (df = 2)	*P*-value	FDR-adjusted *P*-value
Tessellated fundus density	0.06 (0.03, 0.11)	0.07 (0.04, 0.11)	0.09 (0.06, 0.14)	14.421	0.001	0.001
Macular tessellated fundus density	0.04 (0.02, 0.12)	0.09 (0.02, 0.18)	0.16 (0.05, 0.27)	36.262	<0.001	0.001
Optic disc and macular tessellated fundus density	0.09 (0.04, 0.16)	0.12 (0.05, 0.20)	0.19 (0.10, 0.26)	28.456	<0.001	0.001
Peripapillary tessellated fundus density	0.09 (0.03, 0.19)	0.12 (0.06, 0.23)	0.19 (0.11, 0.25)	21.193	<0.001	0.001
Distance from PPA to macula (μm)	3072.90 (2675.76, 4256.00)	3215.45 (2787.14, 4376.09)	2950.32 (2570.59, 4247.91)	12.777	0.002	0.002
PPA arc height (μm)	1449.00 (1148.00, 1960.00)	1652.00 (1316.00, 2114.00)	1890.00 (1463.00, 2317.00)	29.205	<0.001	0.001
Ratio of PPA arc height to vertical disc diameter	0.97 (0.76, 1.16)	1.04 (0.89, 1.15)	1.14 (1.04, 1.31)	32.217	<0.001	0.001
Ratio of PPA area to disc area	0.17 (0.03, 0.40)	0.26 (0.12, 0.45)	0.42 (0.28, 0.72)	14.095	0.001	0.001
PPA arc width (μm)	252.00 (112.00, 420.00)	308.00 (224.00, 448.00)	420.00 (280.00, 616.00)	21.702	<0.001	0.001
Ratio of PPA arc width to horizontal disc diameter	0.15 (0.07, 0.24)	0.19 (0.13, 0.27)	0.26 (0.17, 0.35)	19.494	<0.001	0.001
PPA area (μm^2^)	411796.00 (199773.00, 890967.00)	547820.00 (304000.00, 1063100.00)	945308.00 (524986.00, 1555160.00)	19.921	<0.001	0.001

Patient-specific ocular magnification correction was not applied. Absolute measurements expressed in μm, mm, or μm^2^ should therefore be interpreted with caution.

### Multivariable generalized estimating equation analysis

Multivariable GEE models were constructed to assess the associations of age, sex, axial length, and cataract subtype with selected automated image-derived SLO parameters while accounting for inter-eye correlation. After adjustment for sex, age, axial length, and cataract subtype using multivariable generalized estimating equation models, age remained associated with several tessellated fundus- and peripapillary atrophy-related parameters, including tessellated fundus density, macular tessellated fundus density, optic disc and macular tessellated fundus density, peripapillary tessellated fundus density, and peripapillary atrophy height, width, and area (all nominal *P* < 0.05). Axial length was associated with optic disc structural parameters, including optic disc tilt angle and multiple peripapillary atrophy- and tessellated fundus-related parameters (all nominal *P* < 0.05). Compared with the PSC group, the nuclear and cortical cataract groups remained associated with higher vertical cup-to-disc ratio, larger vertical cup diameter, greater optic disc tilt angle, and larger peripapillary atrophy-related parameters, whereas retinal vessel length was lower in the nuclear and cortical cataract groups (all nominal *P* < 0.05). In addition, sex was associated with selected tessellated fundus-related parameters. Results are shown in Table 4.

**TABLE 4:** Multivariable GEE analysis.

Dependent variable	Independent variable	Beta	95% CI	*P*-value
Vertical cup-to-disc ratio	Nuclear vs. PSC	0.067	0.024, 0.110	0.002
	Cortical vs. PSC	0.063	0.019, 0.106	0.005
Vertical cup diameter (μm)	Nuclear vs. PSC	127.88	36.44, 219.32	0.006
	Cortical vs. PSC	131.12	35.55, 226.70	0.007
Optic disc tilt angle (°)	Nuclear vs. PSC	8.844	1.411, 16.277	0.020
	Cortical vs. PSC	10.004	1.835, 18.172	0.016
	Axial length	−3.462	−6.527, −0.397	0.027
PPA arc height (μm)	Nuclear vs. PSC	530.34	310.15, 750.53	<0.001
	Cortical vs. PSC	516.67	287.70, 745.64	<0.001
	Age	22.87	12.36, 33.37	<0.001
	Axial length	119.41	27.27, 211.55	0.011
PPA arc width (μm)	Nuclear vs. PSC	114.38	43.41, 185.35	0.002
	Cortical vs. PSC	113.63	39.20, 188.07	0.003
	Age	7.74	4.17, 11.32	<0.001
	Axial length	69.26	32.87, 105.65	<0.001
PPA area (μm^2^)	Nuclear vs. PSC	565,709	402,267, 729,151	<0.001
	Cortical vs. PSC	476,621	321,654, 631,587	<0.001
	Age	27,345	13,246, 41,443	<0.001
	Axial length	79,828	15,711, 143,945	0.015
Retinal vessel length (mm)	Nuclear vs. PSC	−186.98	−297.86, −76.10	<0.001
	Cortical vs. PSC	−186.96	−303.52, −70.40	0.002
Tessellated fundus density	Age	0.001	0.000, 0.002	0.001
	Axial length	0.012	0.006, 0.019	<0.001
Macular tessellated fundus density	Female vs. male	−0.028	−0.046, −0.010	0.002
	Age	0.004	0.003, 0.006	<0.001
	Axial length	0.026	0.013, 0.038	<0.001
Optic disc and macular tessellated fundus density	Age	0.003	0.002, 0.005	<0.001
	Axial length	0.024	0.012, 0.037	<0.001
Peripapillary tessellated fundus density	Age	0.003	0.002, 0.004	<0.001
	Axial length	0.022	0.010, 0.035	<0.001

The PPA-area model included 671 eyes from 530 patients because of outcome-specific missing data; the remaining models included 716 eyes from 571 patients. *P*-values are nominal and were not adjusted for multiple testing. Only covariate terms with nominal *P* < 0.05 are shown. The models were exploratory association models. Posterior subcapsular cataract was used as the reference category for cataract subtype; male sex was used as the reference category for sex. Patient-specific ocular magnification correction was not applied. Absolute measurements expressed in μm, mm, or μm^2^ should therefore be interpreted with caution.

### Standardized effect-size analysis

Standardized effect estimates for the 18 selected imaging outcomes are presented in Supplementary Table 5. Among the 14 contrasts involving the seven principal outcomes, the absolute standardized coefficients ranged from 0.258 to 0.619 SD units. Nine contrasts were small in magnitude and five were moderate; none were very small or large according to the descriptive categories. Moderate standardized differences were observed for vertical cup-to-disc ratio in both the nuclear-versus-PSC and cortical-versus-PSC comparisons, PPA arc height in both comparisons, and PPA area in the nuclear-versus-PSC comparison. Standardized differences for vertical cup diameter, optic disc tilt angle, PPA arc width, retinal vessel length, and cortical-versus-PSC PPA area were small. All 14 principal contrasts remained statistically significant after FDR correction. These findings indicate that statistical significance was accompanied by small-to-moderate standardized differences rather than uniformly large effects.

Across all 36 evaluated subtype contrasts, 19 were small, 16 were moderate, and one was large; none were very small. The corrected disc short-to-long axis ratio estimates were moderate for both the nuclear-versus-PSC comparison (standardized β = 0.559) and the cortical-versus-PSC comparison (standardized β = 0.573). The sole comparison meeting the descriptive threshold for a large effect remained the vertical-to-horizontal disc ratio for cortical versus PSC (standardized β = 0.800). These effect-size categories were used only to describe statistical magnitude and should not be interpreted as validated measures of clinical importance.

The age-overlap sensitivity analysis was restricted to patients aged 61–78 years and included 459 patients and 583 eyes: 55 patients/90 eyes with PSC, 253 patients/314 eyes with nuclear cataract, and 151 patients/179 eyes with cortical cataract. After restriction, age distributions did not differ significantly among the three groups (Kruskal–Wallis χ^2^ = 1.159, df = 2, *P* = 0.560).

The directions of all 14 evaluated subtype contrasts were consistent with those obtained in the full-cohort analysis. After adjustment for continuous age, sex, and axial length, the nuclear and cortical cataract groups remained associated with higher vertical cup-to-disc ratio, larger vertical cup diameter, greater PPA arc height, width, and area, and lower retinal vessel length compared with the PSC group after FDR correction. The cortical-versus-PSC association with optic disc tilt angle also remained statistically significant (β = 11.77°, 95% CI 3.387–20.16; FDR-adjusted *P* = 0.008). In contrast, the nuclear-versus-PSC association with optic disc tilt angle was attenuated and was no longer statistically significant (β = 6.508°, 95% CI -1.215 to 14.23; nominal *P* = 0.099). Complete full-cohort and age-overlap estimates are presented in Supplementary Table 4.

### Single-eye sensitivity analysis of multivariable association models

In the single-eye sensitivity analysis, multivariable linear regression models were fitted using the same covariates as the primary GEE models. The direction of the major associations was generally consistent with the primary analyses: nuclear and cortical cataract subtypes remained associated with higher cup-to-disc ratio-related parameters and larger peripapillary atrophy-related parameters compared with PSC, whereas retinal vessel length remained lower in the nuclear and cortical groups. Age remained positively associated with tessellated fundus- and peripapillary atrophy-related parameters. The adjusted R^2^ values ranged from 0.032 to 0.116, indicating that the included variables explained only a limited proportion of between-eye variability. The single-eye analysis was used to assess directional consistency with the clustered-eye analysis and should not be interpreted as a predictive model. No substantial multicollinearity was detected, with all variance inflation factors below 5. Results are summarized in Table 5.

**TABLE 5:** Single-eye sensitivity analysis using multivariable linear regression models.

Dependent variable	Independent variable	Beta	95% CI lower	95% CI upper	*P*-value
Vertical cup-to-disc ratio	Nuclear	0.080	0.047	0.112	<0.001
	Cortical	0.077	0.043	0.111	<0.001
Vertical cup diameter (μm)	Nuclear	156.480	68.971	243.988	<0.001
	Cortical	158.871	66.578	251.164	<0.001
	Sex	60.461	5.765	115.157	0.030
Peripapillary atrophy height (μm)	Nuclear	658.693	450.720	866.667	<0.001
	Cortical	617.806	398.461	837.150	<0.001
	Age	23.757	14.005	33.509	<0.001
Peripapillary atrophy width (μm)	Nuclear	148.675	43.438	201.264	<0.001
	Cortical	141.793	66.864	216.722	<0.001
	Age	8.884	5.553	12.215	<0.001
	Axial length	35.009	12.340	57.678	0.003
Peripapillary atrophy area (μm^2^)	Nuclear	479264.260	101696.623	856831.897	0.013
	Age	29847.337	12394.457	47300.217	<0.001
Retinal vessel length (mm)	Nuclear	−183.199	−228.559	−77.838	<0.001
	Cortical	−182.386	−293.507	−71.264	0.001
	Age	6.465	1.525	11.405	0.010
Tessellated fundus density	Axial length	0.014	0.009	0.018	<0.001
	Age	0.001	0.000	0.002	<0.001
Macular tessellated fundus density	Axial length	0.027	0.017	0.036	<0.001
	Age	0.004	0.003	0.006	<0.001
	Sex	−0.027	−0.045	−0.009	0.004
Optic disc and macular tessellated fundus density	Axial length	0.025	0.017	0.033	<0.001
	Age	0.004	0.002	0.005	<0.001
Peripapillary tessellated fundus density	Axial length	0.023	0.015	0.032	<0.001
	Age	0.003	0.002	0.004	<0.001

Only variables with statistically significant associations are shown. *P*-values are nominal and were not adjusted for multiple testing. The analysis was performed as a sensitivity assessment of directional consistency and was not intended as a predictive model. The models were exploratory association models. Patient-specific ocular magnification correction was not applied. Absolute measurements expressed in μm, mm, or μm^2^ should therefore be interpreted with caution.

## Discussion

This study applied a fixed automated quantitative workflow to analyzable early postoperative SLO images and compared the resulting measurements across clinically recorded cataract subtype groups. Multiple optic disc, cup-to-disc ratio, PPA, tessellated fundus, and vascular parameters differed among subtypes, while age and axial length remained associated with several tessellation- and PPA-related measures in multivariable analyses. Previous studies have reported differences in age distribution, refractive status, and ocular biometry across cataract subtypes (15), which may contribute to the heterogeneity observed here. Accordingly, the present study should be viewed as an exploratory application of a fixed automated quantitative workflow to analyzable postoperative SLO images, with the aim of examining group-level variation. It does not establish subtype-specific structural phenotypes, diagnostic or prognostic performance, clinical decision utility, or causal effects.

In the subtype-based analyses, the nuclear and cortical cataract groups generally showed larger cup-to-disc ratio– and PPA-related measurements than the PSC group, whereas PSC eyes showed greater retinal vessel length. Selected associations persisted after adjustment for age, sex, and axial length. However, cataract subtype may capture differences in age, ocular biometry, refractive status, lens opacity morphology, image-acquisition conditions, and other characteristics rather than directly determine posterior segment morphology. Spherical-equivalent refractive error was not uniformly available and therefore remained an unmeasured potential confounder. This limitation is particularly important when interpreting PPA, tessellated fundus, and retinal vascular measurements and comparisons involving nuclear cataract, because lens-induced myopic refractive change may occur without corresponding axial elongation (16). Adjustment for axial length therefore did not eliminate potential confounding by refractive status. The vessel-length difference also requires caution because it may reflect image-field visibility, segmentation characteristics, or posterior segment morphology rather than a disease-specific vascular phenotype.

The clinical importance of these statistically detectable differences remains uncertain. Standardized effect-size analysis showed that the principal adjusted differences were predominantly small, with moderate effects for vertical cup-to-disc ratio, PPA arc height, and the nuclear-versus-PSC PPA-area comparison (Supplementary Table 5). No large standardized effects were observed among the principal outcomes. These estimates indicate that the findings were not uniformly negligible, but standardized coefficients quantify statistical magnitude rather than clinical benefit or harm. Because validated minimum clinically important differences and clinical decision thresholds are unavailable for these automated SLO measurements, and uniform visual-function and quantitative optical coherence tomography correlates were not available, the results should be interpreted as exploratory group-level variation rather than evidence of clinically important abnormalities or subtype-specific management requirements.

Age and axial length were the most consistent clinical correlates of posterior segment variation. Increasing age was associated with greater tessellated fundus density and PPA-related measurements, consistent with previous histologic and imaging evidence linking aging to choroidal thinning, reduced choroidal perfusion, increased fundus tessellation, and parapapillary atrophic change (17–20). Longer axial length was associated with optic disc tilt, PPA, and tessellated fundus parameters, in keeping with previous reports of posterior-pole stretching, optic disc deformation, and parapapillary remodeling in elongated eyes (19, 21, 22). These established age- and biometry-related relationships provide a plausible background for the observed subtype differences and reinforce that covariate adjustment reduces, but does not eliminate, residual confounding.

The age-overlap sensitivity analysis further clarified the PSC comparisons. Restriction to the common age interval removed the detectable age difference among subtype groups, and the directions of all evaluated subtype contrasts remained consistent. Most associations involving cup-to-disc ratio, vertical cup diameter, PPA measurements, and retinal vessel length remained significant after false discovery rate correction (Supplementary Table 4), whereas the nuclear-versus-PSC association with optic disc tilt angle was attenuated and no longer significant. These findings reduce concern that the principal results were driven solely by the younger PSC group, but the restricted sample remained smaller for PSC and residual ocular and systemic confounding cannot be excluded. Moreover, although cup-to-disc ratio and PPA measurements are used in glaucoma assessment, they were analyzed here only as morphometric image features. Patients with known glaucoma or treated ocular hypertension were excluded, but no standardized glaucoma evaluation or longitudinal follow-up was performed; therefore, the observed differences should not be interpreted as evidence of differential glaucoma risk.

AI-assisted fundus analysis has enabled automated measurements in retinal and optic nerve research (23, 24). The present study shows that an existing automated platform could generate quantitative outputs from analyzable early postoperative SLO images. It did not evaluate diagnostic accuracy, clinical decision support, treatment selection, or outcome prediction. The single-eye sensitivity analysis showed directions and magnitudes broadly consistent with the primary GEE models, reducing concern that the principal findings were driven solely by inter-eye clustering. Nevertheless, the low adjusted R^2^ values indicate that most variation remained unexplained and was likely related to unmeasured ocular, refractive, systemic, surgical, image-quality, or algorithm-related factors. The results therefore support further methodological validation rather than clinical implementation.

Several limitations should be considered. This was a single-center retrospective study, and selection bias and limited generalizability cannot be excluded. The PSC group was smaller than the other groups, and although age-overlap restriction improved comparability, it reduced precision and could not eliminate residual confounding. Cataract subtype was based on retrospective clinical records rather than standardized LOCS III grading. Imaging at approximately 7 days after surgery may have been influenced by subtle corneal change, residual inflammation, pupillary variation, fixation difficulty, early macular change, or surgical characteristics, while exclusion of poor-quality images may itself have introduced selection bias. EVisionAI was not independently revalidated against manual annotations in this cohort; review by a single grader identified gross segmentation failure but did not establish measurement accuracy or subtle segmentation reliability. Spherical-equivalent refractive error was not uniformly available and remained an unmeasured potential confounder. This limitation is particularly important when interpreting PPA, tessellated fundus, and retinal vascular measurements and comparisons involving nuclear cataract, because lens-induced refractive change may not be represented by axial length alone. Patient-specific ocular magnification correction was also unavailable; axial-length adjustment addressed statistical confounding but did not correct image scale, so absolute linear and area measurements may contain scale-related error. Finally, standardized effect estimates do not establish clinical relevance, and the study was not designed to define minimum clinically important differences or individual-level predictive utility.

## Conclusion

This exploratory cross-sectional study applied automated quantitative analysis to analyzable early postoperative SLO images and identified group-level differences across clinically recorded cataract subtype groups. These differences may reflect non-standardized subtype assignment, unmeasured spherical-equivalent refractive error, age, axial length, image characteristics, and other confounding and do not establish subtype-specific structural phenotypes, clinically important abnormalities, or diagnostic, prognostic, or clinical decision utility. Prospective studies incorporating standardized cataract grading, comprehensive refractive and biometric data, cohort-specific algorithm validation, ocular magnification correction, quantitative OCT, and postoperative visual outcomes are required before clinical application can be considered.

## Data Availability

The original contributions presented in this study are included in this article/Supplementary material, further inquiries can be directed to the corresponding authors.

## References

[1] Bourne RRA Flaxman SR Braithwaite T Cicinelli MV Das A Jonas JB et al. Magnitude, temporal trends, and projections of the global prevalence of blindness and distance and near vision impairment: a systematic review and meta-analysis. *The Lancet Global Health*. (2017) 5:e888–97. 10.1016/s2214-109x(17)30293-0 28779882

[2] GBD. 2019 Blindness and vision impairment collaborators; vision loss expert group of the global burden of disease study. causes of blindness and vision impairment in 2020 and trends over 30 years, and prevalence of avoidable blindness in relation to VISION 2020: the right to sight: an analysis for the global burden of disease study. *Lancet Glob. Health* (2021) 9:e144–60. 10.1016/S2214-109X(20)30489-7 33275949 PMC7820391

[3] Donaldson K Fernández-Vega-Cueto L Davidson R Dhaliwal D Hamilton R Jackson M et al. Perioperative assessment for refractive cataract surgery. *J Cataract Refract Surg*. (2018) 44:642–53. 10.1016/j.jcrs.2018.02.022 29891157

[4] Huang X Zhang Z Wang J Meng X Chen T Wu Z. Macular assessment of preoperative optical coherence tomography in ageing Chinese undergoing routine cataract surgery. *Sci Rep*. (2018) 8:5103. 10.1038/s41598-018-22807-7 29572456 PMC5865193

[5] Icoz SGG Usta SA Icoz M. post-phacoemulsification examination of intraocular pathologies that could not bedetected by fundus biomicroscopy or oct in eyes with dense cataract: trying to avoid intra- and post-operative complications. *Photodiagnosis Photodyn Ther*. (2025) 54:104696. 10.1016/j.pdpdt.2025.104696 40581196

[6] Burke J Gibbon S Engelmann J Threlfall A Giarratano Y Hamid C et al. SLOctolyzer: fully automatic analysis toolkit for segmentation and feature extracting in scanning laser ophthalmoscopy images. *Transl Vis Sci Technol*. (2024) 13:7. 10.1167/tvst.13.11.7 39514218 PMC11552063

[7] Asbell PA Dualan I Mindel J Brocks D Ahmad M Epstein S. Age-related cataract. *Lancet*. (2005) 365:599–609. 10.1016/S0140-6736(05)17911-2 15708105

[8] Xu Y Ling SG Dong Z Ke X Lu LN Zou HD. [Development and application of a fundus image quality assessment system based on computer vision technology]. *Zhonghua Yan Ke Za Zhi*. (2020) 56:920–7. 10.3760/cma.j.cn112142-20200409-00257 33342118

[9] Zhao L Yanan C Bin J Saiguang L Yanling W. Correlation analysis between retinal vascular morphological parameters and ischemic stroke. *Chin. J. Ocul. Fundus Dis* (2022) 38:1001–5. 10.3760/cma.j.cn511434-20220919-00502

[10] He HL Liu YX Liu H Zhang X Song H Xu TZ et al. Deep learning-enabled vasculometry depicts phased lesion patterns in high myopia progression. *Asia Pac J Ophthalmol*. (2024) 13:100086. 10.1016/j.apjo.2024.100086 39053733

[11] Wang M Zhou X Liu DN Chen J Zheng Z Ling S. Development and validation of a predictive risk model based on retinal geometry for an early assessment of diabetic retinopathy. *Front Endocrinol.* (2022) 13:1033611. 10.3389/fendo.2022.1033611 36479215 PMC9719996

[12] Wang Q Li T Zhang X Zeng Y Yang Y Zhou Y et al. Distinctive imaging characteristics of retinal and cerebral vessels between central and branch retinal vein occlusion by mri and ai-based image analyzer. *Diagnostics.* (2024) 14:267. 10.3390/diagnostics14030267 38337783 PMC10854905

[13] Gong W Zhang B Zhou D Ling S Yang J Chen J et al. Fundus vascular arcades angle reflects choroidal thickness in highly myopic children and adolescents. *Eye*. (2025) 39:1264–9. 10.1038/s41433-025-03604-9 39827236 PMC12043900

[14] Mongan J Moy L Kahn CE. Checklist for artificial intelligence in medical imaging (CLAIM): a guide for authors and reviewers. *Radiol Artif Intell*. (2020) 2:e200029. 10.1148/ryai.2020200029 33937821 PMC8017414

[15] Richter GM Choudhury F Torres M Azen SP Varma R. Risk factors for incident cortical, nuclear, posterior subcapsular, and mixed lens opacities: the Los Angeles Latino eye study. *Ophthalmology*. (2012) 119:2040–7. 10.1016/j.ophtha.2012.05.001 22771048 PMC3464350

[16] Fotedar R Mitchell P Burlutsky G Wang JJ. Relationship of 10-year change in refraction to nuclear cataract and axial length findings from an older population. *Ophthalmology* (2008) 115:1273–8. 10.1016/j.ophtha.2007.11.003 18222002

[17] Wei WB Xu L Jonas JB Shao L Du KF Wang S et al. Subfoveal choroidal thickness: the Beijing Eye Study. *Ophthalmology*. (2013) 120:175–80. 10.1016/j.ophtha.2012.07.048 23009895

[18] Margolis R Spaide RF. A pilot study of enhanced depth imaging optical coherence tomography of the choroid in normal eyes. *Am J Ophthalmol*. (2009) 147:811–5. 10.1016/j.ajo.2008.12.008 19232559

[19] Yamashita T Terasaki H Tanaka M Nakao K Sakamoto T. Relationship between peripapillary choroidal thickness and degree of tessellation in young healthy eyes. *Graefes Arch Clin Exp Ophthalmol*. (2020) 258:1779–85. 10.1007/s00417-020-04644-5 32248408

[20] Terasaki H Yamashita T Yoshihara N Kii Y Tanaka M Nakao K et al. Location of tessellations in ocular fundus and their associations with optic disc tilt. *PLoS One*. (2016) 11:e0156842. 10.1371/journal.pone.0156842 27275584 PMC4898735

[21] Jonas RA Wang YX Yang H Li JJ Xu L Panda-Jonas S et al. Optic disc-fovea distance axial length and parapapillary zones: the beijing eye study. *PLoS One*. (2015) 10:e0138701. 10.1371/journal.pone.0138701 26390438 PMC4577126

[22] Kim TW Kim M Weinreb RN Woo SJ Park KH Hwang JM. Optic disc change with incipient myopia of childhood. *Ophthalmology* (2012) 119:21e–6e. 10.1016/j.ophtha.2011.07.051 21978594

[23] Gulshan V Peng L Coram M Stumpe MC Wu D Narayanaswamy A et al. Development and validation of a deep learning algorithm for detection of diabetic retinopathy in retinal fundus photographs. *JAMA*. (2016) 316:2402–10. 10.1001/jama.2016.17216 27898976

[24] De Fauw J Ledsam JR Romera-Paredes B Nikolov S Tomasev N Blackwell S et al. Clinically applicable deep learning for diagnosis and referral in retinal disease. *Nat Med*. (2018) 24:1342–50. 10.1038/s41591-018-0107-6 30104768

