# Supplementary material for: Automated postoperative SLO quantification across clinically recorded cataract subtype groups: an exploratory cross-sectional study
Source: Front Med (Lausanne). 2026 Sep 10;13:1896907. doi: 10.3389/fmed.2026.1896907 (PMC13602298; doi:10.3389/fmed.2026.1896907)
Supplement: Supplementary file 1 [file Data_Sheet_1.docx]

**Figure S1. Study flow diagram.** A total of 821 early postoperative SLO images from patients with age-related cataract were initially available during the study period. After exclusion of 105 eyes because of poor image quality, incomplete optic disc or macular visualization, or gross automated segmentation failure, 716 eyes from 571 patients were included in the final quantitative SLO analysis. Cataract subtype was classified according to preoperative slit-lamp records. SLO, scanning laser ophthalmoscopy; AI, artificial intelligence. Exclusion reasons were not mutually exclusive.

**Figure S2. Representative successful EVisionAI segmentations and an excluded poor-quality image.**
(A) Original postoperative SLO image of adequate quality. (B) Optic disc and optic cup segmentation. (C) PPA segmentation and dimensional measurements. (D) Tessellated fundus feature detection and predefined analysis regions. (E) Retinal vessel segmentation and skeletonization. (F) Representative image excluded because of severe defocus, incomplete fundus visualization, and imaging artifacts that prevented reliable automated segmentation. Insets in panels B and C provide magnified views of the corresponding segmentation results. AI-generated outputs were reviewed for gross segmentation failure before statistical analysis; no manual correction was performed. PPA, peripapillary atrophy; SLO, scanning laser ophthalmoscopy.

Supplementary Table S1. Pairwise comparisons of automated image-derived SLO parameters across cataract subtype groups

| automated image-derived parameters | Nuclear cataract | Cortical cataract | PSC | Overall FDR-adjusted P value | Nuclear vs Cortical, adjusted P value | Nuclear vs PSC, adjusted P value | Cortical vs PSC, adjusted P value |
| --- | --- | --- | --- | --- | --- | --- | --- |
| Vertical cup-to-disc ratio | 0.55 (0.48, 0.61) | 0.54 (0.48, 0.60) | 0.54 (0.46, 0.60) | 0.011 | > 0.999 | 0.004 | 0.010 |
| Vertical cup diameter, μm | 896.00 (700.00, 1106.00) | 854.00 (686.00, 1127.00) | 770.00 (539.00, 1008.00) | 0.011 | > 0.999 | 0.006 | 0.010 |
| Cup area, μm² | 649152.00 (399007.00, 946582.00) | 630336.00 (372596.00, 1032630.00) | 465010.00 (234250.00, 780250.00) | 0.034 | > 0.999 | 0.052 | 0.036 |
| Disc tilt angle (°) | 30.86 (13.14, 47.91) | 33.72 (14.86, 50.42) | 18.10 (2.25, 41.89) | 0.034 | > 0.999 | 0.049 | 0.032 |
| PPA arc height, μm | 1638.00 (1291.50, 2114.00) | 1596.00 (1288.00, 2044.00) | 1526.00 (969.50, 2247.00) | 0.003 | > 0.999 | < 0.001 | < 0.001 |
| PPA arc width, μm | 308.00 (224.00, 476.00) | 336.00 (196.00, 448.00) | 280.00 (168.00, 392.00) | 0.045 | > 0.999 | 0.039 | 0.106 |
| PPA area, μm² | 555464.00 (318843.00, 1085542.00) | 615636.00 (307426.00, 992934.00) | 245000.00 (153136.00,460894.00) | 0.003 | 0.749 | < 0.001 | < 0.001 |
| Vessel length, mm | 1460.41 (1257.44, 1837.72) | 1518.85 (1238.08, 1868.14) | 1676.58 (1299.22, 2035.37) | 0.008 | > 0.999 | 0.004 | 0.005 |

Note: Values are presented as median (interquartile range). Pairwise comparisons were performed using estimated marginal means from generalized estimating equation models, with adjustment for multiple testing. PSC, posterior subcapsular cataract; PPA, peripapillary atrophy; SLO, scanning laser ophthalmoscopy; FDR, false discovery rate.

Supplementary Table S2. Distribution of excluded postoperative SLO images by cataract subtype

| **Cataract subtype** | **Initially available SLO images, n** | **Excluded images, n** | **Final included eyes, n** | **Exclusion rate, %** |
| --- | --- | --- | --- | --- |
| Nuclear cataract | 452 | 58 | 394 | 12.8 |
| Cortical cataract | 252 | 35 | 217 | 13.9 |
| PSC | 117 | 12 | 105 | 10.3 |
| Total | 821 | 105 | 716 | 12.8 |

Supplementary Table S3. Comparison of automated image-derived SLO parameters among cataract subtype groups

| **Variable** | **Nuclear (n=394)** | **Cortical (n=217)** | **PSC (n=105)** | **Wald χ² (df=2)** | **P value** | **FDR-adjusted P value** |
| --- | --- | --- | --- | --- | --- | --- |
| Vertical cup-to-disc ratio | 0.55 (0.48, 0.61) | 0.54 (0.48, 0.60) | 0.54 (0.46, 0.60) | 10.414 | 0.005 | 0.011 |
| Area cup-to-disc ratio | 0.29 (0.23, 0.37) | 0.29 (0.23, 0.37) | 0.29 (0.21, 0.36) | 10.023 | 0.007 | 0.011 |
| Vertical cup diameter (μm) | 896.00 (700.00, 1106.00) | 854.00 (686.00, 1127.00) | 770.00 (539.00, 1008.00) | 9.934 | 0.007 | 0.011 |
| Cup area (μm²) | 649152.00 (399007.00, 946582.00) | 630336.00 (372596.00, 1032630.00) | 465010.00 (234250.00, 780250.00) | 6.905 | 0.032 | 0.034 |
| Horizontal cup diameter (μm) | 924.00 (714.00, 1120.00) | 896.00 (672.00, 1155.00) | 868.00 (686.00,  1134.00) | 9.183 | 0.010 | 0.015 |
| Cup circularity | 0.84 (0.80, 0.88) | 0.84 (0.79, 0.88) | 0.82 (0.78, 0.87) | 12.438 | 0.002 | 0.005 |
| Vertical-to-horizontal disc ratio | 0.97 (0.93, 1.02) | 0.98 (0.93, 1.04) | 0.94 (0.88, 1.02) | 22.630 | <0.001 | 0.003 |
| Disc short-to-long axis ratio | 0.89 (0.85, 0.94) | 0.90 (0.86, 0.93) | 0.89 (0.85, 0.91) | 7.661 | 0.022 | 0.027 |
| Disc tilt angle (°) | 30.86 (13.14, 47.91) | 33.72 (14.86, 50.42) | 18.10 (2.25, 41.89) | 7.030 | 0.030 | 0.034 |
| Maximum cup-to-disc rim distance | 1366.63 (1181.32,  1726.32) | 1403.71 (1153.88, 1770.35) | 1274.69 (1134.78,  1748.71) | 7.844 | 0.020 | 0.026 |
| Disc circularity | 0.86 (0.81, 0.90) | 0.86 (0.82, 0.89) | 0.85 (0.81, 0.89) | 7.541 | 0.023 | 0.027 |
| Horizontal cup-to-disc ratio | 0.55 (0.48, 0.62) | 0.54 (0.47, 0.61) | 0.53 (0.46, 0.61) | 11.794 | 0.003 | 0.006 |
| Peripapillary atrophy (PPA) arc height (μm) | 1638.00 (1291.50, 2114.00) | 1596.00 (1288.00, 2044.00) | 1526.00 (969.50, 2247.00) | 19.416 | <0.001 | 0.003 |
| Ratio of PPA arc height to vertical disc diameter | 1.04 (0.88, 1.18) | 1.03 (0.84, 1.15) | 0.99 (0.67, 1.21) | 25.601 | <0.001 | 0.003 |
| PPA arc width (μm) | 308.00 (224.00, 476.00) | 336.00 (196.00, 448.00) | 280.00 (168.00,  392.00) | 6.181 | 0.045 | 0.045 |
| Ratio of PPA arc width to horizontal disc diameter | 0.19 (0.13, 0.27) | 0.19 (0.11, 0.28) | 0.17 (0.10, 0.26) | 7.792 | 0.019 | 0.026 |
| PPA area (μm²) | 555464.00 (318843.00, 1085542.00) | 615636.00 (307426.00, 992934.00) | 245000.00 (153136.00,460894.00) | 23.014 | <0.001 | 0.003 |
| Vessel length (mm) | 1460.41 (1257.44, 1837.72) | 1518.85 (1238.08, 1868.14) | 1676.58 (1299.22, 2035.37) | 10.845 | 0.004 | 0.008 |

Note: PSC, posterior subcapsular cataract.

Supplementary Table S4. Comparison of full-cohort and age-overlap generalized estimating equation estimates for selected subtype-associated SLO measurements

| **Outcome** | **Comparison** | **Full cohort β (95% CI)** | **Full cohort**  **nominal P** | **Age-overlap β (95% CI)** | **Age-overlap nominal P** | **Age-overlap FDR-adjusted P** | **Direction consistent** |
| --- | --- | --- | --- | --- | --- | --- | --- |
| Vertical cup-to-disc ratio | Nuclear vs PSC | 0.067 (0.024, 0.110) | 0.002 | 0.077 (0.030, 0.125) | 0.001 | 0.003 | Yes |
| Vertical cup-to-disc ratio | Cortical vs PSC | 0.063 (0.019, 0.106) | 0.005 | 0.074 (0.025, 0.122) | 0.003 | 0.005 | Yes |
| Vertical cup diameter (μm) | Nuclear vs PSC | 127.88 (36.44, 219.32) | 0.006 | 167.34 (67.75, 266.92) | <0.001 | 0.003 | Yes |
| Vertical cup diameter (μm) | Cortical vs PSC | 131.12 (35.55, 226.70) | 0.007 | 167.07 (63.21, 270.94) | 0.002 | 0.003 | Yes |
| Optic disc tilt angle (°) | Nuclear vs PSC | 8.844 (1.411, 16.277) | 0.020 | 6.508 (-1.215, 14.23) | 0.099 | 0.099 | Yes |
| Optic disc tilt angle (°) | Cortical vs PSC | 10.004 (1.835, 18.172) | 0.016 | 11.77 (3.387, 20.16) | 0.006 | 0.008 | Yes |
| PPA arc height (μm) | Nuclear vs PSC | 530.34 (310.15, 750.53) | <0.001 | 567.37 (336.84, 797.90) | <0.001 | <0.001 | Yes |
| PPA arc height (μm) | Cortical vs PSC | 516.67 (287.70, 745.64) | <0.001 | 522.34 (282.62, 762.06) | <0.001 | <0.001 | Yes |
| PPA arc width (μm) | Nuclear vs PSC | 114.38 (43.41, 185.35) | 0.002 | 116.27 (39.60, 192.93) | 0.003 | 0.005 | Yes |
| PPA arc width (μm) | Cortical vs PSC | 113.63 (39.20, 188.07) | 0.003 | 105.51 (26.68, 184.35) | 0.009 | 0.010 | Yes |
| PPA area (μm²) | Nuclear vs PSC | 565,709 (402,267, 729,151) | <0.001 | 562,753 (380,048, 745,457) | <0.001 | <0.001 | Yes |
| PPA area (μm²) | Cortical vs PSC | 476,621 (321,654, 631,587) | <0.001 | 477,355 (307,284, 647,425) | <0.001 | <0.001 | Yes |
| Retinal vessel length (mm) | Nuclear vs PSC | -186.98 (-297.86, -76.10) | <0.001 | -158.49 (-275.04, -41.95) | 0.008 | 0.010 | Yes |
| Retinal vessel length (mm) | Cortical vs PSC | -186.96 (-303.52, -70.40) | 0.002 | -152.37 (-273.90, -30.84) | 0.014 | 0.015 | Yes |

Note: The age-overlap sensitivity analysis was restricted to patients aged 61–78 years, corresponding to the intersection of the subtype-specific 5th–95th percentile age ranges. Because of outcome-specific missing data, the age-overlap PPA-area models included 580 eyes from 458 patients, whereas the remaining age-overlap models included 583 eyes from 459 patients: PSC, 55 patients/90 eyes; nuclear cataract, 253 patients/314 eyes; and cortical cataract, 151 patients/179 eyes. Age distributions did not differ significantly among the three groups after restriction (Kruskal–Wallis χ²=1.159, df=2, P=0.560). Estimates were obtained using multivariable generalized estimating equation models adjusted for continuous age, sex, and axial length, with patient identification as the clustering variable and an exchangeable working correlation structure. PSC and male sex were used as reference categories. Full-cohort P values are nominal. For the age-overlap analysis, nominal P values and Benjamini–Hochberg FDR-adjusted P values across the 14 evaluated subtype contrasts are reported. Patient-specific ocular magnification correction was not applied; absolute linear and area measurements should therefore be interpreted cautiously. CI, confidence interval; FDR, false discovery rate; PPA, peripapillary atrophy; PSC, posterior subcapsular cataract; SLO, scanning laser ophthalmoscopy.

Supplementary Table S5. Standardized effect estimates for subtype-related differences in automated SLO measurements

| **Outcome** | **Comparison** | **Original-unit β (95% CI)** | **Standardized β (95% CI)** | **Effect-size magnitude** | **Nominal P value** | **FDR-adjusted P value** |
| --- | --- | --- | --- | --- | --- | --- |
| Vertical cup-to-disc ratio | Nuclear vs PSC | 0.067 (0.024, 0.110) | 0.551 (0.200, 0.902) | Moderate | 0.002 | 0.004 |
|  | Cortical vs PSC | 0.063 (0.019, 0.106) | 0.514 (0.159, 0.869) | Moderate | 0.005 | 0.008 |
| Area cup-to-disc ratio | Nuclear vs PSC | 0.046 (0.014, 0.078) | 0.420 (0.132, 0.709) | Small | 0.004 | 0.008 |
|  | Cortical vs PSC | 0.041 (0.008, 0.073) | 0.373 (0.077, 0.669) | Small | 0.013 | 0.016 |
| Vertical cup diameter (μm) | Nuclear vs PSC | 127.88 (36.44, 219.32) | 0.395 (0.112, 0.677) | Small | 0.006 | 0.010 |
|  | Cortical vs PSC | 131.12 (35.55, 226.70) | 0.405 (0.110, 0.700) | Small | 0.007 | 0.011 |
| Cup area (μm²) | Nuclear vs PSC | 119,107 (-1,326, 239,539) | 0.250 (-0.003, 0.502) | Small | 0.053 | 0.053 |
|  | Cortical vs PSC | 149,580 (16,534, 282,627) | 0.314 (0.035, 0.593) | Small | 0.028 | 0.028 |
| Horizontal cup diameter (μm) | Nuclear vs PSC | 121.16 (25.23, 217.10) | 0.356 (0.074, 0.637) | Small | 0.013 | 0.016 |
|  | Cortical vs PSC | 129.35 (27.68, 231.02) | 0.380 (0.081, 0.678) | Small | 0.013 | 0.016 |
| Cup circularity | Nuclear vs PSC | 0.100 (0.045, 0.154) | 0.780 (0.350, 1.209) | Moderate | <0.001 | 0.001 |
|  | Cortical vs PSC | 0.096 (0.041, 0.151) | 0.751 (0.320, 1.183) | Moderate | <0.001 | 0.002 |
| Vertical-to-horizontal disc ratio | Nuclear vs PSC | 0.121 (0.054, 0.189) | 0.692 (0.308, 1.076) | Moderate | <0.001 | 0.001 |
|  | Cortical vs PSC | 0.140 (0.074, 0.207) | 0.800 (0.422, 1.179) | Large | <0.001 | <0.001 |
| Disc short-to-long axis ratio | Nuclear vs PSC | 0.054 (0.013, 0.094) | 0.559 (0.137, 0.981) | Moderate | 0.009 | 0.013 |
|  | Cortical vs PSC | 0.055 (0.015, 0.095) | 0.573 (0.155, 0.992) | Moderate | 0.007 | 0.011 |
| Optic disc tilt angle (°) | Nuclear vs PSC | 8.844 (1.411, 16.277) | 0.258 (0.041, 0.474) | Small | 0.020 | 0.021 |
|  | Cortical vs PSC | 10.004 (1.835, 18.172) | 0.291 (0.053, 0.529) | Small | 0.016 | 0.018 |
| Maximum cup-to-disc rim distance | Nuclear vs PSC | 154.08 (25.19, 282.97) | 0.373 (0.061, 0.685) | Small | 0.019 | 0.021 |
|  | Cortical vs PSC | 174.22 (40.11, 308.32) | 0.422 (0.097, 0.746) | Small | 0.011 | 0.014 |
| Disc circularity | Nuclear vs PSC | 0.054 (0.014, 0.094) | 0.570 (0.149, 0.992) | Moderate | 0.008 | 0.011 |
|  | Cortical vs PSC | 0.054 (0.014, 0.094) | 0.570 (0.150, 0.991) | Moderate | 0.008 | 0.011 |
| Horizontal cup-to-disc ratio | Nuclear vs PSC | 0.070 (0.027, 0.113) | 0.558 (0.215, 0.901) | Moderate | 0.001 | 0.004 |
|  | Cortical vs PSC | 0.067 (0.023, 0.110) | 0.532 (0.186, 0.878) | Moderate | 0.003 | 0.005 |
| PPA arc height (μm) | Nuclear vs PSC | 530.34 (310.15, 750.53) | 0.619 (0.362, 0.876) | Moderate | <0.001 | <0.001 |
|  | Cortical vs PSC | 516.67 (287.70, 745.64) | 0.603 (0.336, 0.870) | Moderate | <0.001 | <0.001 |
| Ratio of PPA arc height to vertical disc diameter | Nuclear vs PSC | 0.338 (0.213, 0.463) | 0.719 (0.453, 0.986) | Moderate | <0.001 | <0.001 |
|  | Cortical vs PSC | 0.324 (0.195, 0.453) | 0.689 (0.415, 0.963) | Moderate | <0.001 | <0.001 |
| PPA arc width (μm) | Nuclear vs PSC | 114.38 (43.41, 185.35) | 0.402 (0.152, 0.651) | Small | 0.002 | 0.004 |
|  | Cortical vs PSC | 113.63 (39.20, 188.07) | 0.399 (0.138, 0.660) | Small | 0.003 | 0.005 |
| Ratio of PPA arc width to horizontal disc diameter | Nuclear vs PSC | 0.078 (0.036, 0.119) | 0.449 (0.209, 0.690) | Small | <0.001 | 0.001 |
|  | Cortical vs PSC | 0.080 (0.036, 0.125) | 0.464 (0.208, 0.720) | Small | <0.001 | 0.001 |
| PPA area (μm²) | Nuclear vs PSC | 565,709 (402,267, 729,151) | 0.564 (0.401, 0.727) | Moderate | <0.001 | <0.001 |
|  | Cortical vs PSC | 476,621 (321,654, 631,587) | 0.475 (0.321, 0.630) | Small | <0.001 | <0.001 |
| Retinal vessel length (mm) | Nuclear vs PSC | -186.98 (-297.86, -76.10) | -0.454 (-0.722, -0.185) | Small | <0.001 | 0.003 |
|  | Cortical vs PSC | -186.96 (-303.52, -70.40) | -0.453 (-0.736, -0.171) | Small | 0.002 | 0.004 |

Note: Original-unit and standardized coefficients were obtained from outcome-specific multivariable generalized estimating equation models adjusted for continuous age, sex, axial length, and clinically recorded cataract subtype. Patient identification was specified as the clustering variable, with an exchangeable working correlation structure and robust sandwich standard errors. Standardized outcomes were calculated using the mean and standard deviation of the full analytic cohort for each outcome. PSC and male sex were the reference categories. Benjamini-Hochberg FDR correction was applied across the 36 evaluated subtype contrasts. Effect-size categories were used only as descriptive statistical guides: very small, |beta|<0.20; small, 0.20 to <0.50; moderate, 0.50 to <0.80; and large, >=0.80. Missing data were handled using outcome-specific complete-case analysis. Cup area and PPA area models included 671 eyes from 530 patients; all remaining models included 716 eyes from 571 patients. Patient-specific ocular magnification correction was not applied. CI, confidence interval; FDR, false discovery rate; PPA, peripapillary atrophy; PSC, posterior subcapsular cataract; SLO, scanning laser ophthalmoscopy.
