# Supplementary material for: Automated postoperative SLO quantification across clinically recorded cataract subtype groups: an exploratory cross-sectional study
Source: Front Med (Lausanne). 2026 Sep 10;13:1896907. doi: 10.3389/fmed.2026.1896907 (PMC13602298; doi:10.3389/fmed.2026.1896907)
Supplement: Supplementary file 2 [file Data_Sheet_2.docx]

**Checklist**

| **Item No.** | **Recommendation** | **Location in manuscript** | **Reported Information** |
| --- | --- | --- | --- |
| 1(a) | Indicate the study design with a commonly used term in the title or abstract. | Title; Abstract | The title identifies the study as a “Retrospective Cross-Sectional Imaging Study.” The abstract describes the work as a retrospective observational imaging study including patients with age-related cataract. |
| 1(b) | Provide an informative and balanced summary of what was done and what was found. | Abstract | The abstract summarizes the objective, study population, imaging method, cataract subtype classification, AI-derived SLO parameters, statistical methods, main findings, and exploratory conclusion. |
| 2 | Explain the scientific background and rationale for the investigation. | Introduction | The manuscript explains that age-related cataract may limit preoperative fundus visualization and that postoperative SLO combined with AI-based image analysis may help quantify posterior segment structural features after removal of lens opacity. |
| 3 | State specific objectives, including any prespecified hypotheses. | Abstract; Introduction | The study aimed to compare AI-derived SLO fundus structural parameters among nuclear, cortical, and posterior subcapsular cataract subtypes and to explore associations of age, axial length, and cataract subtype with quantitative fundus imaging features. |
| 4 | Present key elements of study design early in the paper. | Methods—Study design and participants | The manuscript states that this was a retrospective observational imaging study of cataract surgery patients. |
| 5 | Describe the setting, locations, and relevant dates. | Methods—Study design and participants | The study was conducted at the Affiliated Eye Hospital of Shandong University of Traditional Chinese Medicine. Eligible patients underwent phacoemulsification with intraocular lens implantation between August 2023 and August 2025. |
| 6(a) | Give the eligibility criteria and the sources and methods of participant selection. | Methods—Study design and participants | Inclusion criteria included age-related cataract, age ≥50 years, no family history of hereditary ocular disease, available routine ophthalmic data, and fundus imaging data suitable for quantitative analysis. Exclusion criteria included non-age-related cataract, glaucoma/ocular hypertension history, prior retinal surgery, retinal vascular occlusion, diabetic retinopathy, age-related macular degeneration, pathologic myopia, inability to cooperate with examination, incomplete records, or poor-quality images. |
| 7 | Clearly define all outcomes, exposures, predictors, potential confounders, and effect modifiers. | Methods—Study design and participants; AI-based fundus image analysis; Statistical analysis | The grouping variable was cataract subtype: nuclear, cortical, or posterior subcapsular cataract. Outcomes were AI-derived SLO parameters, including tessellated fundus-related parameters, optic disc morphology, PPA-related parameters, and retinal vascular parameters. Main covariates included age, sex, axial length, and cataract subtype. |
| 8 | For each variable of interest, give sources of data and details of methods of assessment. | Methods—Ophthalmic examinations and surgery; AI-based fundus image analysis | Cataract subtype was based on preoperative slit-lamp records. Axial length was obtained using IOLMaster biometry. BCVA was recorded as decimal visual acuity. SLO images were obtained approximately 7 days after surgery. EVisionAI was used for automated localization, segmentation, and quantitative extraction of fundus parameters. |
| 9 | Describe any efforts to address potential sources of bias. | Methods; Discussion—Limitations | Poor-quality images and gross segmentation failures were excluded before statistical analysis. AI-generated outputs were reviewed by a trained ophthalmic grader. GEE models accounted for inter-eye correlation. A single-eye sensitivity analysis was performed. Limitations acknowledge selection bias, smaller PSC group, retrospective cataract subtype classification, postoperative-only imaging, and lack of independent EVisionAI revalidation in this cohort. |
| 10 | Explain how the study size was arrived at. | Methods—AI-based fundus image analysis; Results; Supplementary Figure S1 | The study included available eligible cases during the study period. Of 821 initially available postoperative SLO images, 105 were excluded because of poor image quality, incomplete optic disc or macular visualization, or gross AI segmentation failure, leaving 716 eyes from 571 patients for final analysis. |
| 11 | Explain how quantitative variables were handled in the analyses. | Methods—Statistical analysis | Continuous variables were summarized as mean ± standard deviation or median with interquartile range, depending on distribution. Age was categorized into ≤69, 70–79, and ≥80 years for age-stratified analyses. AI-derived imaging parameters were descriptively reported as median with interquartile range. |
| 12(a) | Describe all statistical methods, including those used to control for confounding. | Methods—Statistical analysis | Patient-level baseline characteristics were compared using chi-square/Fisher’s exact test, ANOVA, or Kruskal–Wallis H test, as appropriate. Eye-level AI-derived SLO parameters were analyzed using GEE models with patient identification as the clustering variable. Multivariable GEE models were adjusted for age, sex, axial length, and cataract subtype. |
| 12(b) | Describe any methods used to examine subgroups and interactions. | Methods—Statistical analysis; Results | Analyses were performed across cataract subtype groups and age categories. Formal interaction analyses were not reported. |
| 12(c) | Explain how missing data were addressed. | Methods—Statistical analysis | Missing data were handled by complete-case analysis. |
| 12(d) | Describe any sensitivity analyses. | Methods—Statistical analysis; Results—Single-eye sensitivity analysis | A single-eye sensitivity analysis was performed using multivariable linear regression models because only one eye per patient was included. The first operated eye was selected; if both eyes were operated on during the same period, one eye was randomly selected. |
| 13(a) | Report numbers of individuals at each stage of the study. | Methods; Results; Supplementary Figure S1 | A total of 821 early postoperative SLO images were initially available. After exclusion of 105 images, 716 eyes from 571 patients were included in the final analysis. The final cataract subtype groups included 394 nuclear cataract eyes, 217 cortical cataract eyes, and 105 PSC eyes. |
| 13(b) | Give reasons for non-participation or exclusion at each stage. | Methods—AI-based fundus image analysis; Supplementary Figure S1; Supplementary Table S2 | Images were excluded because of poor image quality, incomplete optic disc or macular visualization, or gross AI segmentation failure. Exclusion rates across cataract subtypes are reported in Supplementary Table S2. |
| 13(c) | Consider use of a flow diagram. | Supplementary Figure S1 | A study flow diagram is provided to show the initial image set, excluded images, final analytic dataset, and cataract subtype groups. |
| 14(a) | Give characteristics of study participants. | Results—Baseline characteristics; Table 1 | Baseline characteristics include sex, age, axial length, and BCVA across nuclear, cortical, and PSC groups. |
| 14(b) | Indicate number of participants with missing data for each variable of interest. | Methods—Statistical analysis | Complete-case analysis was used. No additional missing data were present among the variables included in the final complete-case analysis. |
| 15 | Report numbers of outcome events or summary measures. | Results; Tables 2–5; Supplementary Tables S1–S3 | The study reports summary measures for AI-derived SLO parameters across cataract subtype groups and age groups. Representative subtype comparisons are shown in Table 2, age-group comparisons in Table 3, multivariable GEE results in Table 4, and single-eye sensitivity analysis in Table 5. Pairwise comparisons and complete AI-derived parameters are provided in supplementary tables. |
| 16(a) | Give unadjusted and adjusted estimates and their precision. | Results; Tables 2–5 | Group comparisons report medians with interquartile ranges, Wald χ² statistics, P values, and FDR-adjusted P values. Multivariable GEE and single-eye sensitivity analyses report regression coefficients, 95% confidence intervals, and P values. |
| 16(b) | Report category boundaries when continuous variables were categorized. | Methods—Statistical analysis; Table 3 | Age was categorized into ≤69 years, 70–79 years, and ≥80 years. |
| 17 | Report other analyses performed, including subgroup and sensitivity analyses. | Results; Supplementary Tables | The manuscript reports age-stratified analyses, pairwise comparisons for selected representative parameters, multivariable GEE models, and single-eye sensitivity analysis. |
| 18 | Summarize key results with reference to study objectives. | Discussion | The manuscript states that AI-assisted postoperative SLO imaging revealed measurable posterior segment structural heterogeneity among cataract subtypes and age groups. |
| 19 | Discuss limitations, including potential sources of bias or imprecision. | Discussion—Limitations | Limitations include single-center retrospective design, selection bias, smaller PSC group, lack of functional retinal assessments and long-term visual outcomes, postoperative-only imaging, possible early postoperative image effects, retrospective cataract subtype classification without standardized LOCS III grading, and lack of independent EVisionAI revalidation against manual annotations in this cohort. |
| 20 | Give a cautious overall interpretation considering objectives, limitations, multiplicity, and other evidence. | Discussion; Conclusion | Findings are interpreted as exploratory imaging associations rather than evidence of causality, subtype-specific posterior segment disease, validated preoperative prediction, or prognostic utility. |
| 21 | Discuss generalizability. | Discussion; Conclusion | The manuscript states that prospective multicenter studies incorporating ocular biometry, standardized cataract grading, image quality control, AI validation metrics, and postoperative visual outcomes are needed to clarify clinical utility. |
| 22 | Give the source of funding and role of funders. | Funding section | Funding sources are listed, including the Special Clinical Research Project at Shandong University of Chinese Medicine, the Key R&D Program of Shandong Province, and the Traditional Chinese Medicine Science and Technology Project of Shandong Province. The role of the funders is not explicitly stated. The funders had no role in the study design, data collection, data analysis, interpretation of results, manuscript preparation, or decision to submit the manuscript for publication. |
